# Metabolic Characteristics of a Novel Ultrasound Quantitative Diagnostic Index for Nonalcoholic Fatty Liver Disease

**DOI:** 10.1038/s41598-019-44453-3

**Published:** 2019-05-28

**Authors:** Yin-Yin Liao, Chih-Kuang Yeh, Kuo-Chin Huang, Po-Hsiang Tsui, Kuen-Cheh Yang

**Affiliations:** 1 Department of Biomedical Engineering, Hungkuang University, Taichung, Taiwan; 2 Department of Biomedical Engineering and Environmental Sciences, National Tsing Hua University, Hsinchu, Taiwan; 3 Department of Family Medicine, College of Medicine, National Taiwan University, Taipei, Taiwan; 4 Department of Family Medicine, National Taiwan University Hospital, Taipei, Taiwan; 5 Department of Family Medicine, National Taiwan University Hospital, BeiHu Branch, Taipei, Taiwan; 6 Department of Medical Imaging and Intervention, Chang Gung Memorial Hospital at Linkou, Taoyuan, Taiwan; 7 Department of Medical Imaging and Radiological Sciences, College of Medicine, Chang Gung University, Taoyuan, Taiwan; 8 Medical Imaging Research Center, Institute for Radiological Research, Chang Gung University and Chang Gung Memorial Hospital at Linkou, Taoyuan, Taiwan

**Keywords:** Metabolic syndrome, Biomedical engineering, Ultrasonography

## Abstract

Nonalcoholic fatty liver disease (NAFLD) is an emerging epidemic worldwide and is regarded as a hepatic manifestation of metabolic syndrome (MetS). Only a few studies have discussed the biological features associated with quantitative assessment of ultrasound for characterizing NAFLD. Our aim was to delineate relevant metabolic characteristics using a new quantitative tool, the ultrasound quantitative diagnostic index (QDI). A total of 394 ultrasound data were analyzed to extract texture-feature parameters, the signal-to-noise ratio (SNR), and the slope of the center frequency downshift (CFDS) for determining the QDI. The texture index, SNR, and CFDS slope were all negatively correlated with high-density lipoprotein and positively correlated with other anthropometric indices and metabolic factors (all *P* < 0.05). The SNR had the greatest contribution to anthropometric and biochemical factors, followed by the texture index and CFDS slope. An increase in 1 unit of QDI score engendered a 9% higher risk of MetS, reflecting that the tool is feasible for use in identifying MetS (area under the receiver operating characteristic curve: 0.89). The QDI was correlated with metabolic factors and an independent predictor for MetS. Thus, this QDI might be a feasible method for use in clinical surveillance, epidemiology research, and metabolic function evaluations in patients with NAFLD.

## Introduction

Nonalcoholic fatty liver disease (NAFLD), characterized by excess triglyceride (TG) accumulation within hepatocytes, is considered to be the most common chronic liver disease. NAFLD encompasses a spectrum of diseases ranging from simple steatosis to steatohepatitis, advanced cirrhosis, and hepatocellular carcinoma^1^. Furthermore, NAFLD has been linked to metabolic abnormalities including abdominal obesity, diabetes mellitus, hypertension, and hyperlipidemia, and it is a hepatic manifestation of metabolic syndrome (MetS)^2,3^.

Although the histologic severity of NAFLD is associated with some components of MetS, the liver biopsy procedure is invasive and can cause sampling errors and severe complications^4–6^. Therefore, a liver biopsy is not appropriate for the evaluation or follow-up of NAFLD in usual clinical care. Increasing numbers of studies, such as large-scale screenings for NAFLD, have noted the need for a convenient and reliable surveillance tool^7–10^. Specifically, a noninvasive and accessible method of quantifying liver fat content would be useful in research and in the clinical field. Magnetic resonance spectroscopy (MRS) has emerged as a reliable means of identifying metabolic disorders^11,12^. Nevertheless, MRS is a relatively costly and time-consuming procedure. Ultrasound is accepted as a tool for initial NAFLD screening because of its widespread availability and safety. Several prediction models based on MetS features have been proposed for ultrasound diagnosis of NAFLD^13–16^. However, applications of ultrasound for NAFLD classification are limited by visual inspection and operator dependency, which result in only fair agreement with MetS components.

Quantitative assessments of ultrasound findings have been investigated with the aim of producing an objective method for diagnosing NAFLD. Analyses of ultrasound B-mode image texture statistics and ultrasound radiofrequency signals have been widely used to classify liver abnormalities^17–20^. Within the extensive literature on quantitative ultrasound diagnosis of NAFLD, little research has focused on the relationships between metabolic factors, liver function tests, and quantitative ultrasound parameters.

**Figure 1:**
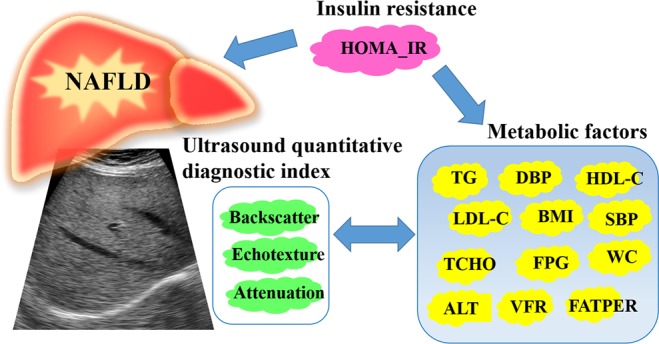
Interrelationship between metabolic syndrome and ultrasound quantitative diagnostic index used for non-alcoholic fatty liver disease (NAFLD) detection. Abbreviations: BMI: body mass index; WC: waist circumference; SBP: systolic blood pressure; DBP: diastolic blood pressure; FATPER: fat percentage; VFR: visceral fat rating; FPG: fasting plasma glucose; TCHO: total cholesterol; TG: triglycerides; HDL-C: high-density lipoprotein cholesterol; LDL-C: low-density lipoprotein cholesterol; ALT: alanine aminotransferase; HOMA-IR: homeostasis model assessment of insulin resistance.

In our previous study, we developed a new ultrasound quantitative diagnostic index (QDI) for detecting small changes in the characteristics of liver fat and enhancing the means of grading NAFLD severity^21^. Because NAFLD and MetS exhibit common interactions and pathogenic mechanisms^22,23^, the current study was conducted to explore the correlations between the parameters of QDI and metabolic findings (Fig. 1). The quantitative ultrasound parameters representing biological manifestations could be used in clinical surveillance and epidemiology to reflect the nature of metabolic disarrangement in NAFLD.

## Materials and Methods

### Participants

All voluntary participants were recruited from Hsinchu City, Taiwan, and asked to complete a standardized questionnaire that elicited information concerning the exclusion criteria of excessive alcohol use (>20 g/day for women and >30 g/day for men) and chronic liver disease (including chronic hepatitis carrier, autoimmune, drug-induced, vascular and inherited hemochromatosis, and Wilson disease). In total, 394 individuals older than 20 years were enrolled in this study. Informed written consent was obtained from all participants. Physical examination, anthropometric measurements, biochemical blood analyses, and abdominal ultrasound were performed on the same day. The study was conducted in accordance with the Declaration of Helsinki, and the study protocol was approved by the Institutional Review Board of National Taiwan University Hospital (approval number, IRB#201210012RIC).

### Anthropometric indices and biochemical analyses

Routine physical examinations were used to collect anthropometric and metabolic data. Body mass index (BMI) was calculated as weight (kg) divided by height (m) squared. Waist circumference (WC) was measured midway between the costal margin and iliac crests. Values of systolic blood pressure (SBP) and diastolic blood pressure (DBP) were recorded (mmHg). Fasting plasma glucose (FPG), total cholesterol (TCHO), high-density lipoprotein cholesterol (HDL-C), low-density lipoprotein cholesterol (LDL-C), alanine aminotransferase (ALT), and TG were examined after participants had fasted for 8 hours overnight. Body fat percentage (FATPER) and visceral fat (visceral fat rating, VFR, with range 1–59) were assessed using bioelectrical impedance analysis (Tanita MC980). Insulin resistance was measured using the homeostasis model assessment of insulin resistance (HOMA-IR) developed by the Diabetes Trials Unit at the Oxford Center for Diabetes, Endocrinology, and Metabolism (avail- able at http://www.dtu.ox.ac.uk/homacalculator).

### Definition of MetS

MetS was defined according to the modified National Cholesterol Education Program Adult Treatment Panel III Criteria, which have been used in numerous published studies of the Taiwanese population. Diagnosis required fulfillment of at least three of the following criteria: (1) WC ≥90 cm in men and ≥80 cm in women; (2) SBP ≥130 mmHg, DBP ≥85 mmHg, or use of medications for hypertension; (3) hyperglycemia (FPG ≥5.5 mmol/L) or use of medications for diabetes; (4) hypertriglyceridemia (TG ≥1.69 mmol/L) or use of medications for hyperlipidemia; and (5) low HDL-C (≤0.45 mmol/L in men and ≤0.56 mmol/L in women).

### Abdominal ultrasound scanning

Three research physicians with more than 20 years of experience performed the ultrasound sonography procedures in this study. All physicians determined and followed the ultrasound scanning protocol used to acquire images of the liver. A portable clinical ultrasound scanner (Model 3000, Terason, Burlington, MA, USA) equipped with a 3.5-MHz central frequency convex-array transducer (Model 5C2A, Terason) was used as a system platform. The transducer contained 128 elements and had a pulse length of approximately 2.3 mm. The instrument settings were standardized for the imaging of all participants. For each patient, a standard abdominal ultrasound examination was performed. Additionally, the subcostal scanning approach was used to perform quantitative analysis because of avoiding any bowel or ribs shadowing over the liver. Then, the liver section was selected by the expert physician so as contain only liver parenchyma with no major blood vessels.

The software kit provided by Terason was used to acquire the raw data comprised 128 scan lines of backscattered signals at a sampling rate of 30 MHz. The backscattered signals were demodulated using the absolute value of the Hilbert transform to obtain the envelope image. A B-mode image at a dynamic range of 40 dB was formed by the log-compressed envelope image. MATLAB software (MATLAB, version R2015b; Mathworks, Natick, Mass) was used in image processing and quantitative analyses.

### Ultrasound QDI

Texture-feature parameters, the signal-to-noise ratio (SNR), and the slope of the center frequency downshift (CFDS) were combined to produce the QDI. The texture-feature parameters were extracted based on a gray level co-occurrence matrix (GLCM) and represented the quantified homogeneity and heterogeneity of the B-mode image^21^. GLCM obtained three texture-feature parameters which uncorrelated and have typical characteristics of texture analysis, that is autocorrelation (AC), sum average (SA), and sum variance (SV). The AC was a measure of gray level linear dependencies in the B-mode image. The SA and SV quantified the mean and extent of the gray level sum histogram, respectively; the former indicated the homogeneous brightness of the B-mode image, and the latter described the dispersion (with regard to the mean) of the B-mode image. We incorporated linear discriminant analysis (LDA) algorithm to implement a linear combination of the three texture-feature parameters and generate the LDA-texture index. The LDA-texture index described the degree of coarse liver echotexture.

The SNR was defined as the root mean square of the ultrasound envelope divided by the root mean square of the noise^21^. The SNR quantified the degree of arrangement and concentration in scatterers; it increased as a liver echogenicity becomes brighter. The CFDS was determined as the ratio of the estimated center frequency and the center frequency of the transducer^21^. The estimated center frequency was the midpoint of the power spectral distribution, and it was calculated from the full width at half maximum in the power spectrum. The ultrasound CFDS image was formed by moving small windows around each pixel area to obtain local CFDS values until the entire envelope image has been scanned. Linear regression was applied to measure the CFDS slope in the ultrasound CFDS image, and the diffraction was neglected with assuming the constant slope of CFDS along the selected depth. The slope of CFDS was approximately proportional to the attenuation coefficient because of a linear correlation between the center frequency and attenuation coefficient. The CFDS slope represented posterior attenuation of the deep liver parenchyma. The tertiles of the LDA-texture index, SNR, and CFDS slope were scored as 0, 1, and 2, respectively. Their scores were then summed to determine the total QDI, which ranged from 0 to 6. For additional details, see the description of our recent study^21^.

### Statistical analysis

Categorical data are presented as percentages, and continuous variables are presented as mean ± standard deviation. Trend analysis and the Cochran–Armitage trend test were used to test for trends in the continuous and categorical variables among the tertiles of the LDA-texture index, SNR, and CFDS slope. The correlation between each ultrasound quantitative parameter and the anthropometric and metabolic factors was assessed using the Pearson correlation coefficient (*r*). To assess the individual contribution of the three parameters for the metabolic factors, we established a multiple regression model with the LDA-texture index, SNR, and CFDS slope as independent covariates and the metabolic factors as dependent variables. The regression coefficient (*β*) of each quantitative parameter represented its contribution to the metabolic factors. Moreover, the association between MetS and the QDI was measured using a multiple logistic regression model with adjustments for age, sex, smoking, alcohol consumption, betel nut chewing, exercise time per week, menopause (women only), and HOMA-IR. Ability to distinguish MetS was assessed by the areas under the receiver operating characteristic curves (AUCs). A probability value (*P*) of <0.05 was considered statistically significant. SAS software (SAS Inc., version 9.3, Cary, NC, USA) was utilized for statistical analyses.

## Results

In total, 394 participants comprising 151 men and 243 women aged 40.5 ± 11.3 years (mean ± standard deviation) were recruited. They were middle adult (40.5 ± 11.3 yrs) with overweight (BMI = 24.1 ± 4.6). Supplementary Table 1 showed the details of anthropometric and metabolic values of all participants. The characteristics of the participants were classified into tertiles according to the LDA-texture, SNR, and CFDS slope (Table 1). The participants’ age was not significantly associated with the LDA-texture index or CFDS slope but was significantly associated with the SNR (*P* = 0.0028). The observed BMI, WC, SBP, DBP, FATPER, VFR, FPG, TCHO, TG, LDL-C, HOMA-IR, and ALT increased with the LDA-texture, SNR, and CFDS slope, whereas the HDL-C decreased (all *P* for trend < 0.05).

**Table 1:** Characteristics of participants in different tertiles of ultrasound quantitative parameters.

Variables	LDA-texture index			*P*	SNR			*P*	CFDS slope			*P*
	T1	T2	T3		T1	T2	T3		T1	T2	T3	
N	131	133	130		130	133	131		130	133	131	
Men	25%	34%	56%	<0.0001	21%	42%	52%	<0.0001	25%	35%	54%	<0.0001
Age (yrs)	40.6	40.8	40.1	0.5627	38.3	40.9	42.4	0.0028	41.2	40.7	39.7	0.246
BMI (kg/m^2^)	22.1	23.8	26.5	<0.0001	21.4	24.4	26.5	<0.0001	22.7	23.4	26.3	<0.0001
WC (cm)	76.8	81.6	87.4	<0.0001	75.1	82.1	88.3	<0.0001	77.8	80.3	87.5	<0.0001
SBP (mmHg)	115.0	122.3	130.2	<0.0001	115.4	120.8	131.3	<0.0001	118.6	120.2	128.7	<0.0001
DBP (mmHg)	73.2	77.4	83.1	<0.0001	73.4	77.2	83.1	<0.0001	75.2	76.7	81.8	0.0003
FATPER (%)	27.5	29.0	30.3	0.0083	26.4	29.3	31.2	<0.0001	28.4	28.3	30.2	0.1967
VFR	5.8	7.5	10.2	<0.0001	4.8	8.4	10.3	<0.0001	6.3	7.1	10.0	<0.0001
FPG (mmol/L)	4.70	4.74	5.16	0.0002	4.59	4.84	5.16	<0.0001	4.68	4.72	5.20	<0.0001
TCHO (mmol/L)	4.90	4.94	5.15	0.0602	4.66	5.20	5.12	0.0009	4.83	4.97	5.18	0.0185
TG (mmol/L)	0.92	1.27	1.61	<0.0001	0.86	1.36	1.59	<0.0001	0.90	1.09	1.82	<0.0001
HDL-C (mmol/L)	1.61	1.52	1.31	<0.0001	1.62	1.50	1.32	<0.0001	1.61	1.52	1.32	<0.0001
LDL-C (mmol/L)	3.01	3.02	3.35	0.0035	2.79	3.27	3.31	<0.0001	2.92	3.13	3.33	0.0018
HOMA_IR	0.88	1.16	1.42	0.0002	0.92	1.11	1.41	0.0002	0.88	1.13	1.46	<0.0001
ALT (µkat/L)	0.32	0.38	0.63	<0.0001	0.30	0.44	0.60	<0.0001	0.32	0.41	0.60	<0.0001
MetS	7%	17%	31%	<0.0001	6%	14%	52%	<0.0001	9%	8%	38%	<0.0001

^a^Abbreviations: ALT: alanine aminotransferase; BMI: body mass index; CFDS: center frequency downshift; DBP: diastolic blood pressure; FATPER: fat percentage; FPG: fasting plasma glucose; HDL-C: high-density lipoprotein cholesterol; HOMA-IR: homeostasis model assessment of insulin resistance; LDA-texture: linear discriminant analysis was applied to combine the texture features; LDL-C: low-density lipoprotein cholesterol; MetS: metabolic syndrome; SBP: systolic blood pressure; SNR: signal-to-noise ratio; TCHO: total cholesterol; TG: triglycerides; VFR: visceral fat rating; WC: waist circumference.

^b^T1: tertile 1; T2: tertile 2; T3: tertile 3.

The Pearson correlation coefficients determined for the correlation of the ultrasound quantitative parameters with the metabolic factors are presented in Table 2. The LDA-texture, SNR, and CFDS slope were all negatively correlated with HDL-C and positively correlated with BMI, WC, SBP, DBP, FATPER, VFR, FPG, TCHO, TG, LDL-C, HOMA-IR, and ALT (all *P* < 0.05). The LDA-texture index exhibited good correlation with BMI (*r* = 0.42), WC (*r* = 0.41), SBP (*r* = 0.40), VFR (*r* = 0.40), and ALT (*r* = 0.41). Additionally, the SNR was strongly correlated with BMI (*r* = 0.45), WC (*r* = 0.48), SBP (*r* = 0.41), VFR (*r* = 0.49), and TG (*r* = 0.41). The CFDS slope exhibited a stronger correlation with TG (*r* = 0.27) than with the other metabolic factors.

**Table 2:** Pearson correlation coefficient (𝛾) between metabolic factors and ultrasound quantitative parameters.

Variables	LDA-texture index	SNR	CFDS slope
	*r*	*r*	*r*
BMI (kg/m^2^)	0.42^***^	0.45^***^	0.21^***^
WC (cm)	0.41^***^	0.48^***^	0.22^***^
SBP (mmHg)	0.40^***^	0.41^***^	0.24^***^
DBP (mmHg)	0.37^***^	0.35^***^	0.18^***^
FATPER (%)	0.16^**^	0.23^***^	0.10^*^
VFR	0.40^***^	0.49^***^	0.20^***^
FPG (mmol/L)	0.21^***^	0.25^***^	0.14^**^
TCHO (mmol/L)	0.14^**^	0.21^***^	0.19^***^
TG^#^ (mmol/L)	0.37^***^	0.41^***^	0.27^***^
HDL-C^#^ (mmol/L)	−0.33^***^	−0.28^***^	−0.15^**^
LDL-C (mmol/L)	0.20^***^	0.25^***^	0.20^***^
HOMA-IR^#^	0.31^***^	0.32^***^	0.24^***^
ALT (µkat/L)	0.41^***^	0.34^***^	0.24^***^

^a#^Variable was managed by logarithmic transformation.

^b^*r*: Pearson correlation coefficient.

^c^*P < 0.05; ^**^P < 0.01; ^***^P < 0.001.

^d^Abbreviations: ALT: alanine aminotransferase; BMI: body mass index; CFDS: center frequency downshift; DBP: diastolic blood pressure; FATPER: fat percentage; FPG: fasting plasma glucose; HDL-C: high-density lipoprotein cholesterol; HOMA-IR: homeostasis model assessment of insulin resistance; LDA-texture: linear discriminant analysis was applied to combine the texture features; LDL-C: low-density lipoprotein cholesterol; SBP: systolic blood pressure; SNR: signal-to-noise ratio; TCHO: total cholesterol; TG: triglycerides; VFR: visceral fat rating; WC: waist circumference.

The contributions of the combined LDA-texture, SNR, and CFDS slope to each metabolic factor were determined according to the regression coefficient (*β*) in the multiple regression model (Table 3). The SNR significantly contributed to all metabolic factors (all *P* < 0.01). The LDA-texture index had significant effects on all metabolic factors except for FATPER, FPG, TCHO, and LDL-C. The CFDS slope exhibited no significant contributions to most of the metabolic factors (except for TCHO, TG, LDL-C, and HOMA-IR).

**Table 3:** Contributions of combined multiple ultrasound quantitative parameters for various metabolic factors.

Variables	LDA-texture index	SNR	CFDS slope
	*β*_1_	*β*_2_	*β*_3_
BMI (kg/m^2^)	5.38^***^	7.22^***^	0.34
WC (cm)	11.67^***^	19.67^***^	1.80
SBP (mmHg)	18.09^***^	21.81^***^	5.48
DBP (mmHg)	14.37^***^	12.99^***^	0.82
FATPER (%)	2.02	7.28^***^	1.17
VFR	4.76^***^	8.70^***^	−0.01
FPG (mmol/L)	0.46	0.89^***^	0.25
TCHO (mmol/L)	−0.05	0.80^**^	0.72^**^
TG^#^ (mmol/L)	0.53^***^	0.86^***^	0.37^*^
HDL-C^#^ (mmol/L)	−0.32^***^	−0.21^**^	0.01
LDL-C (mmol/L)	0.22	0.78^***^	0.56^*^
HOMA-IR^#^	0.45^*^	0.71^***^	0.47^*^
ALT (µkat/L)	0.48^***^	0.32^***^	0.13

^a#^Variable was managed by logarithmic transformation.

^b*^P < 0.05; ^**^P < 0.01; ^***^P < 0.001.

^c^Abbreviations: ALT: alanine aminotransferase; BMI: body mass index; CFDS: center frequency downshift; DBP: diastolic blood pressure; FATPER: fat percentage; FPG: fasting plasma glucose; HDL-C: high-density lipoprotein cholesterol; HOMA-IR: homeostasis model assessment of insulin resistance; LDA-texture: linear discriminant analysis was applied to combine the texture features; LDL-C: low-density lipoprotein cholesterol; SBP: systolic blood pressure; SNR: signal-to-noise ratio; TCHO: total cholesterol; TG: triglycerides; VFR: visceral fat rating; WC: waist circumference.

The associations between the QDI and the risk of MetS determined using the multiple logistic regression models are presented in Table 4. In model 1, a higher QDI was correlated with a higher risk of MetS after adjustment for age and sex [odds ratio (OR): 1.75, 95% confidence interval (CI): 1.46–2.09, *P* < 0.0001]. In model 2, after further adjustment for smoking, alcohol consumption, betel nut chewing, exercise time per week, and menopause, the OR determined using the QDI for MetS was 1.12 (95% CI: 1.09–1.16, *P* < 0.0001). In model 3, after further adjustment for HOMA-IR, the OR determined using the QDI for MetS was 1.09 (95% CI: 1.06–1.13, *P* < 0.001). The corresponding values of the AUCs used to distinguish MetS were 0.79 (95% CI: 0.73–0.85), 0.88 (95% CI: 0.83–0.92), and 0.89 (95% CI: 0.84–0.93).

**Table 4:** Risk of metabolic syndrome according to the quantitative diagnostic index level.

	OR (95% CI)	AUCs (95% CI)
Model 1	1.75 (1.46–2.09)^*^	0.79 (0.73–0.85)
Model 2	1.12 (1.09–1.16)^*^	0.88 (0.83–0.92)
Model 3	1.09 (1.06–1.13)^*^	0.89 (0.84–0.93)

^a^Model 1: adjusted for age and sex.

^b^Model 2: model 1 plus further adjustment for smoking, alcohol consumption, betel nut chewing, exercise time per week and menopause (women only).

^c^Model 3: model 2 plus further adjustment for HOMA-IR; OR for HOMA-IR: 2.07 (95% CI: 1.35–3.17, *P* = 0.0008).

^d*^P < 0.001.

^e^Abbreviations: AUCs: areas under the receiver operating characteristic curves; CI: confidence interval; OR: odds ratio.

## Discussion

Our previous study demonstrated that the QDI increases significantly with the severity of NAFLD^21^. In the present study, liver fat amount quantified using the QDI was significantly associated with MetS. The risk of MetS was 1.09 (OR:1.09, 95% CI: 1.06–1.13) times greater in individuals with high QDI values than it was in those with low QDI values after adjustment for age, sex, smoking, alcohol consumption, betel nut chewing, exercise time per week, menopause, and HOMA-IR. These findings are consistent with those reported regarding the relationship between ultrasound-diagnosed NAFLD and metabolic derangements in previous studies^13–16^. However, most of these studies have only discriminated between pathological and normal livers based on the presence of a bright liver echo pattern and have not graded NAFLD severity. A few studies have used an ultrasound scoring system to diagnose NAFLD severity^24–26^. The semiquantitative liver ultrasound scoring system presented by Hamaguchi *et al*. was significantly correlated with MetS (OR: 1.37, 95% CI: 1.26–1.49), but the analysis was not adjusted for lifestyle factors or insulin resistance^24^. Yang *et al*. showed an association between increasing NAFLD severity, as assessed using the semiquantitative ultrasonographic fatty liver indicator score, and a greater risk of MetS (OR: 1.4, 95% CI: 1.2–1.6) after adjustment for BMI and insulin resistance^26^. However, the inclusion of subjective determinations is a disadvantage of the aforementioned semiquantitative scoring system. The QDI is a relatively objective measure and reflects useful information regarding various acoustic characteristics of liver tissue.

Quantitative ultrasound techniques represent a reliable adjunct approach for NAFLD diagnoses, offering a valuable secondary assessment for physicians. Badawi *et al*. presented a computerized tissue characterization employing eight quantitative features of ultrasound images to differentiate diffuse liver disease and achieve sensitivity of 96%^27^. Acharya *et al*. combined ultrasound image texture, higher order spectra, and discrete wavelet transform to distinguish between normal and fatty livers, obtaining an accuracy of 93.3%^28^. Lin *et al*. used ultrasound backscatter coefficient values to identify patients with NAFLD in training and testing groups, achieving 94% and 88% accuracy rates, respectively^29^. Many published studies have focused on improving diagnostic accuracy for NAFLD using quantitative ultrasound techniques. Nevertheless, additional studies are required to determine the correlation between quantitative ultrasound parameters and metabolic, anthropometric, and biochemical factors in order to emphasize the potential applications of functional ultrasound. The present study attempted to supplement the literature.

For the separate predictions of BMI, WC, SBP, FATPER, VFR, FPG, TCHO, TG, LDL-C, and HOMA-IR, the SNR had a higher *β* coefficient than did the LDA-texture index and CFDS slope. In brief, the SNR exhibited the greatest influence on these biochemical factors. Among the three variables, the LDA-texture index had the highest *β* coefficients in predicting DBP, HDL-C, and ALT. However, the CFDS slope had higher *β* coefficients in predicting TCHO, LDL-C, and HOMA-IR than did the LDA-texture index. These results indicate that the SNR exhibited the greatest contribution to the prediction of biochemical factors, followed by the LDA-texture index and CFDS slope.

The SNR can depict the arrangement and distribution of scatters caused by the concentration of fatty droplets in liver tissue^21,30^. The findings of the present study reveal that the SNR exhibited the strongest association with BMI, WC, SBP, FATPER, VFR, FPG, TCHO, TG, LDL-C, and HOMA-IR. BMI, WC, FATPER, and VFR are associated with body fat or visceral fat^31,32^. TCHO, TG, and LDL-C are nonpolar lipid substances that are escorted through the blood vessels by lipoproteins^33–35^. Finally, HOMA-IR is used to quantify insulin resistance, and insulin is a critical regulator of virtually all aspects of adipocyte biology^36,37^. In other words, the SNR is closely correlated not only with the distribution of liver fat deposits but also with body fat, visceral fat, and blood lipid levels. The LDA-texture index can be used to describe microstructural and macrostructural changes within the liver parenchyma for quantification of liver tissue heterogeneity^17,21,38^. The results of the present study indicate that the LDA-texture index had strong predictive power in DBP, HDL-C, and ALT. HDL-C is often referred to as good cholesterol, because HDL particles help remove fats and cholesterol from cells and deliver them to the liver for excretion, thereby playing a paramount role in the reverse cholesterol transport mechanism^39–41^. ALT is usually measured clinically in diagnostic evaluations of hepatocellular injury^42,43^. Several studies have reported that low HDL-C is associated with abnormal ALT levels^44,45^. Therefore, the LDA-texture index may be associated with hepatocellular function (e.g., hepatic inflammation). The CFDS slope mirrors the composition and biochemical environment of the liver^19,21,46^. In the present study, the CFDS slope was a significant variable for predicting the observed TCHO, TG, LDL-C, and HOMA-IR. TCHO, TG, LDL-C, are associated with lipid levels, signifying that hepatocyte lipid accumulation could be a predominant influence in the CFDS slope.

Both the SNR and CFDS slope were relevant to lipid profiles (e.g., TCHO, TG, and LDL-C), and the SNR had a higher contribution to blood lipids than did the CFDS slope. However, the CFDS slope remains indispensable in grading NAFLD severity, because the SNR and CFDS slope may provide information on different ultrasound physical characteristics to describe liver fat distribution. Although increased blood lipid abnormality could reflect altered liver function, this metric may not be sufficiently sensitive to accurately reflect liver fat accumulation. Based on the widely recognized relationship between liver fat and MetS^23,26^, we demonstrated that the QDI score was significantly correlated with and could be used to identify MetS. This finding demonstrates that quantitative ultrasound imaging methods are critical in diagnosing NAFLD.

The QDI remains limited by some factors. The choice of tertile cutoff points for the LDA-texture index, SNR, and CFDS slope was based on the distribution of the sample. The values of the LDA-texture index, SNR, and CFDS slope may change with different ultrasound systems, but these estimates obtained from different ultrasound systems still show the same trends. The values of the CFDS slope were probably affected by estimation errors related to the analytical methods and the influence of diffraction effect, resulting in relatively poor correlation with anthropometric and biochemical findings. The histologic analysis was not usable as a diagnostic reference for assessing liver fat. Furthermore, ultrasound scanning is operator-dependent. Thus, additional large-scale clinical trials should be performed to establish the QDI and facilitate its acceptance in the medical community.

Several studies have demonstrated that NAFLD is an independent risk factor for MetS. In most of these studies, NAFLD was diagnosed through simple dichotomous (yes vs. no) diagnosis using ultrasound. Our research revealed that our proposed quantitative parameters correlated with metabolic factors in NAFLD. Isolated hepatic steatosis is not entirely benign^47^. For example, the amount of liver fat is strongly associated with cardiovascular disease, cancer, and many extrahepatic diseases^48^. Simple steatosis may directly evolve into hepatocellular carcinoma^49^. Additionally, the essentiality of liver biopsy is disputed^4–6^. Therefore, critical demand remains for an accessible and simple quantification tool in clinical and epidemiology fields. Several clinical trials regarding pharmacotherapy for NAFLD are in progress^50^. Although liver biopsy procedures are necessary in these clinical trials, a reliable quantitative surveillance tool should be available for practical applications. In sum, the proposed tool can be used for quantitative measurements that may not only assist clinicians in monitoring disease status but also help patients understand and follow their treatment course.

The primary findings of this study reveal that the QDI parameters predict MetS and significantly correlate with anthropometric and biochemical factors. The QDI may be a promising modality for use in clinical surveillance, epidemiology research, and metabolic function evaluation in patients with NAFLD. Some quantitative analysis approaches have also been used for the diagnosis of hepatic steatosis and demonstrated to correlate with several MetS components, such as controlled attenuation parameter, acoustic structure quantification, Kurtosis coefficient, and entropy^51–54^. These approaches reflect different physical properties of the liver to characterize liver tissue microstructure^55^. With the development of inflammation and fibrosis biomarkers, combining the QDI parameters with other ultrasound features would become a functional evaluation parameter for NAFLD and MetS. Ultrasound contrast agents have been used to not only improve ultrasound imaging quality but also for differentiating none or mild from severe fibrosis in NAFLD patients^56–58^. Compared with NAFLD patients, contrast enhancement is decreased in patients with non-alcoholic steatohepatitis^59^. Therefore, the QDI would be a valuable measurement in contrast-enhanced ultrasound for evaluating the diagnosis of hepatic manifestation of MetS. Future studies could examine the use of multiple QDI parameters in a larger cohort of participants and in longitudinal follow-up of patients with NAFLD.

## Supplementary information

Supplementary Table 1

## References

[1] Yu AS, Keeffe EB (2002). Nonalcoholic fatty liver disease. Rev. Gastroenterol. Disord..

[2] Leite NC, Salles GF, Araujo AL, Villela-Nogueira CA, Cardoso CR (2009). Prevalence and associated factors of nonalcoholic fatty liver disease in patients with type-2 diabetes mellitus. Liver Int..

[3] Fabbrini E, Sullivan S, Klein S (2010). Obesity and nonalcoholic fatty liver disease: biochemical, metabolic, and clinical implications. Hepatology.

[4] Sumida Y, Nakajima A, Itoh Y (2014). Limitations of liver biopsy and non-invasive diagnostic tests for the diagnosis of nonalcoholic fatty liver disease/nonalcoholic steatohepatitis. World J. Gastroenterol..

[5] Tapper EB, Lok AS (2017). Use of liver imaging and biopsy in clinical practice. N. Engl. J. Med..

[6] Castera L (2018). Diagnosis of non-alcoholic fatty liver disease/non-alcoholic steatohepatitis: Non-invasive tests are enough. Liver Int..

[7] Mehta SR, Thomas EL, Bell JD, Johnston DG, Taylor-Robinson SD (2008). Non-invasive means of measuring hepatic fat content. World J. Gastroenterol..

[8] Machado MV, Cortez-Pinto H (2013). Non-invasive diagnosis of non-alcoholic fatty liver disease. A critical appraisal. J. Hepatol..

[9] Siegert S (2013). Diagnosing fatty liver disease: a comparative evaluation of metabolic markers, phenotypes, genotypes and established biomarkers. PLoS One.

[10] Marcellin P, Kutala BK (2018). Liver diseases: a major, neglected global public health problem requiring urgent actions and large-scale screening. Liver Int..

[11] Szczepaniak LS (2005). Magnetic resonance spectroscopy to measure hepatic triglyceride content: prevalence of hepatic steatosis in the general population. Am. J. Physiol. Endocrinol. Metab..

[12] Ducluzeau PH (2013). MRI measurement of liver fat content predicts the metabolic syndrome. Diabetes Metab..

[13] Bedogni G (2006). The Fatty Liver Index: a simple and accurate predictor of hepatic steatosis in the general population. BMC Gastroenterol..

[14] Hamaguchi M (2012). Identification of individuals with non-alcoholic fatty liver disease by the diagnostic criteria for the metabolic syndrome. World J. Gastroenterol..

[15] Kim NH (2014). Clinical and metabolic factors associated with development and regression of nonalcoholic fatty liver disease in nonobese subjects. Liver Int..

[16] Yang BL (2015). External validation of fatty liver index for identifying ultrasonographic fatty liver in a large-scale cross-sectional study in Taiwan. PLoS One.

[17] Gaitini D (2004). Feasibility study of ultrasonic fatty liver biopsy: texture vs. attenuation and backscatter. Ultrasound Med. Biol..

[18] Xia MF (2012). Standardized ultrasound hepatic/renal ratio and hepatic attenuation rate to quantify liver fat content: an improvement method. Obesity.

[19] Kanayama Y, Kamiyama N, Maruyama K, Sumino Y (2013). Real-time ultrasound attenuation imaging of diffuse fatty liver disease. Ultrasound Med. Biol..

[20] Tsui PH, Wan YL (2016). Effects of fatty infiltration of the liver on the Shannon entropy of ultrasound backscattered signals. Entropy.

[21] Liao YY (2016). Multifeature analysis of an ultrasound quantitative diagnostic index for classifying nonalcoholic fatty liver disease. Sci. Rep..

[22] Fattahi MR, Niknam R, Safarpour A, Sepeh-rimanesh M, Lotfi M (2016). The prevalence of metabolic syndrome in non-alcoholic fatty liver disease; a population-based study. Middle East. J. Dig. Dis..

[23] Sookoian S, Pirola CJ (2016). Nonalcoholic fatty liver disease and metabolic syndrome: shared genetic basis of pathogenesis. Hepatology.

[24] Hamaguchi M (2007). The severity of ultrasonographic findings in nonalcoholic fatty liver disease reflects the metabolic syndrome and visceral fat accumulation. Am. J. Gastroenterol..

[25] Ballestri S (2012). Ultrasonographic fatty liver indicator, a novel score which rules out NASH and is correlated with metabolic parameters in NAFLD. Liver Int..

[26] Yang KC (2016). Association of non-alcoholic fatty liver disease with metabolic syndrome independently of central obesity and insulin resistance. Sci. Rep..

[27] Badawi AM, Derbala AS, Youssef AM (1999). Fuzzy logic algorithm for quantitative tissue characterization of diffuse liver diseases from ultrasound images. Int. J. Med. Inform..

[28] Acharya UR (2012). Data mining framework for fatty liver disease classification in ultrasound: a hybrid feature extraction paradigm. Med. Phys..

[29] Lin SC (2015). Noninvasive diagnosis of nonalcoholic fatty liver disease and quantification of liver fat using a new quantitative ultrasound technique. Clin. Gastroenterol. Hepatol..

[30] Shankar PM (2000). A general statistical model for ultrasonic backscattering from tissues. IEEE Trans. Ultrason. Ferroelec. Freq. Contr..

[31] Jakobsen MU, Berentzen T, Sørensen TI, Overvad K (2007). Abdominal obesity and fatty liver. Epidemiol. Rev..

[32] Ko YH, Wong TC, Hsu YY, Kuo KL, Yang SH (2017). The correlation between body fat, visceral fat, and nonalcoholic fatty liver disease. Metab. Syndr. Relat. Disord..

[33] Spector AA (1984). Plasma lipid transport. Clin. Physiol. Biochem..

[34] Fon-Tacer K, Rozman D (2011). Nonalcoholic Fatty liver disease: focus on lipoprotein and lipid deregulation. J. Lipids.

[35] Tomizawa M (2014). Triglyceride is strongly associated with nonalcoholic fatty liver disease among markers of hyperlipidemia and diabetes. Biomed. Rep..

[36] Marchesini G (1999). Association of nonalcoholic fatty liver disease with insulin resistance. Am. J. Med..

[37] Utzschneider KM, Kahn SE (2006). The role of insulin resistance in nonalcoholic fatty liver disease. J. Clin. Endocrinol. Metab..

[38] Valckx FM, Thijssen JM (1997). Characterization of echographic image texture by co-occurrence matrix parameters. Ultrasound Med. Biol..

[39] Daniels TF, Killinger KM, Michal JJ, Wright RW, Jiang Z (2009). Lipoproteins, cholesterol homeostasis and cardiac health. Int. J. Biol. Sci..

[40] Chatrath H, Vuppalanchi R, Chalasani N (2012). Dyslipidemia in patients with nonalcoholic fatty liver disease. Semin. Liver Dis..

[41] Eren E, Yilmaz N, Aydin O (2012). High density lipoprotein and it’s dysfunction. Open Biochem. J..

[42] Giannini EG, Testa R, Savarino V (2005). Liver enzyme alteration: a guide for clinicians. CMAJ.

[43] Sattar N, Forrest E, Preiss D (2014). Non-alcoholic fatty liver disease. BMJ.

[44] Setji TL (2006). Nonalcoholic steatohepatitis and nonalcoholic Fatty liver disease in young women with polycystic ovary syndrome. J. Clin. Endocrinol. Metab..

[45] Jiang ZG, Mukamal K, Tapper E, Robson SC, Tsugawa Y (2014). Low LDL-C and high HDL-C levels are associated with elevated serum transaminases amongst adults in the United States: a cross-sectional study. PLoS One.

[46] Pauly H, Schwan HP (1971). Mechanism of absorption of ultrasound in liver tissue. J. Acoust. Soc. Am..

[47] Diehl AM, Day C (2017). Cause, pathogenesis, and treatment of nonalcoholic steatohepatitis. N. Engl. J. Med..

[48] Adams LA, Anstee QM, Tilg H, Targher G (2017). Non-alcoholic fatty liver disease and its relationship with cardiovascular disease and other extrahepatic diseases. Gut..

[49] Fan JG, Kim SU, Wong VW (2017). New trends on obesity and NAFLD in Asia. J. Hepatol..

[50] Rotman Y, Sanyal AJ (2017). Current and upcoming pharmacotherapy for non-alcoholic fatty liver disease. Gut..

[51] Mikolasevic I (2016). Factors associated with significant liver steatosis and fibrosis as assessed by transient elastography in patients with one or more components of the metabolic syndrome. J. Diabetes Complic..

[52] Lin YH, Liao YY, Yeh CK, Yang KC, Tsui PH (2018). Ultrasound entropy imaging of nonalcoholic fatty liver disease: association with metabolic syndrome. entropy.

[53] Tsui PH, Wan YL (2016). Effects of Fatty In ltration of the Liver on the Shannon Entropy of Ultrasound Backscattered Signals. Entropy.

[54] Yang Kuen Cheh, Liao Yin-Yin, Tsui Po-Hsiang, Yeh Chih-Kuang (2019). Ultrasound imaging in nonalcoholic liver disease: current applications and future developments. Quantitative Imaging in Medicine and Surgery.

[55] Zhou Z, Zhang Q, Wu W, Wu S, Tsui PH (2019). Hepatic Steatosis Assessment Using Quantitative Ultrasound Parametric Imaging Based on Backscatter Envelope Statistics. Appl Sci.

[56] Zhang K, Chen H, Guo X (2015). Double-scattering/reflection in a single nanoparticle for intensified ultrasound imaging. Sci. Rep..

[57] Nasr P, Hilliges A, Thorelius L, Kechagias S, Ekstedt M (2016). Contrast-enhanced ultrasonography could be a non-invasive method for differentiating none or mild from severe fibrosis in patients with biopsy proven non-alcoholic fatty liver disease. Scand. J. Gastroenterol..

[58] Wang Y, Zhang K, Xua YH, Chen HR (2018). Nanosized hollow colloidal organosilica nanospheres with high elasticity for contrast-enhanced ultrasonography of tumors. ACS Biomater. Sci. Eng..

[59] Iijima H (2007). Decrease in accumulation of ultrasound contrast microbubbles in non-alcoholic steatohepatitis. Hepatol. Res..

