# Supplementary material for: Metabolic Characteristics of a Novel Ultrasound Quantitative Diagnostic Index for Nonalcoholic Fatty Liver Disease
Source: Sci Rep. 2019 May 28;9:7922. doi: 10.1038/s41598-019-44453-3 (PMC6538602; doi:10.1038/s41598-019-44453-3)
Supplement: Supplementary file 1 — Supplementary Table 1 [file 41598_2019_44453_MOESM1_ESM.docx]

**Metabolic Characteristics of a Novel Ultrasound Quantitative Diagnostic Index for Nonalcoholic Fatty Liver Disease**

Yin-Yin Liao^1^, Chih-Kuang Yeh^2^, Kuo-Chin Huang^3,4,5^, Po-Hsiang Tsui^6,7,8^, Kuen-Cheh Yang^,3,5,*^

^1^ Department of Biomedical Engineering, Hungkuang University, Taichung, Taiwan

^2^ Department of Biomedical Engineering and Environmental Sciences, National Tsing Hua University, Hsinchu, Taiwan

^3^ Department of Family Medicine, College of Medicine, National Taiwan University, Taipei, Taiwan

^4^ Department of Family Medicine, National Taiwan University Hospital, Taipei, Taiwan

^5^ Department of Family Medicine, National Taiwan University Hospital, BeiHu Branch, Taipei, Taiwan

^6^ Department of Medical Imaging and Intervention, Chang Gung Memorial Hospital at Linkou, Taoyuan, Taiwan

^7^ Department of Medical Imaging and Radiological Sciences, College of Medicine, Chang Gung University, Taoyuan, Taiwan

^8^ Medical Imaging Research Center, Institute for Radiological Research, Chang Gung University and Chang Gung Memorial Hospital at Linkou, Taoyuan, Taiwan

**Supplementary Table 1. Basic characteristics of participants (N=394)**

| **Variable** | ***N* (%)** | | **Mean ± SD** | | | **Range** | | |
| --- | --- | --- | --- | --- | --- | --- | --- | --- |
| Men | 151 (38.3) |  | | |  | | |  |
| Age (years) |  | 40.5 ± 11.3 | | | (20–72) | | |  |
| **Anthropometric variables** | | | | | | |  |  |
| WC (cm) |  | 81.1 ± 11.2 | | (55–120) | | |  |  |
| BMI (kg/m2) |  | 24.1 ± 4.6 | | (14.9–43.7) | | |  |  |
| SBP (mmHg) |  | 122.5 ± 16.3 | | (86-180) | | |  |  |
| DBP (mmHg) |  | 77.9 ± 11.9 | | (50-133) | | |  |  |
| FATPER (%) |  | 29 ± 7.8 | | (7-58.5) | | |  |  |
| VFR^*^ |  | 7.8 ± 4.7 | | (1-25) | | |  |  |
| **Biochemistry parameters** | | | | | | |  |  |
| FPG (mg/dL) |  | 87.7 ± 17.6 | | (58–272) | | |  |  |
| TCHO (mg/dL) |  | 192.9 ± 35.5 | | (101–320) | | |  |  |
| TG (mg/dL) |  | 112.4 ± 90.3 | | (25–888) | | |  |  |
| HDL-C (mg/dL) |  | 57.3 ± 15.8 | | (25–120) | | |  |  |
| LDL-C (mg/dL) |  | 120.8 ± 32.5 | | (47–248) | | |  |  |
| ALT (IU/L) |  | 26.5 ± 21.5 | | (2-151) | | |  |  |
| Insulin (μU/mL) |  | 9.10 ± 8.16 | | (2–84.4) | | |  |  |
| HOMA-IR |  | 1.17 ± 1.00 | | (0.26–10.2) | | |  |  |
| MetS | 73 (18.5%) |  | |  | | |  |  |

Abbreviations: WC: waist circumference; BMI: body mass index; SBP: systolic blood pressure; DBP: diastolic blood pressure; FATPER: fat percentage; VFR: visceral fat rating; FPG: fasting plasma glucose; TCHO: total cholesterol; TG: triglycerides; HDL-C: high-density lipoprotein cholesterol; LDL-C: low-density lipoprotein cholesterol; ALT: alanine aminotransferase; HOMA-IR: homeostasis model assessment of insulin resistance; MetS: metabolic syndrome

Fat percentage (FATPER) and visceral fat rating (VFR) were measured by bioelectrical impedance analysis (Tanita-MC980)

^*^VFR range 1-59
